# Enhancing and Improving Resident Doctor Handover Practices at Black Country NHS Foundation Trust

**DOI:** 10.1192/bjo.2025.10370

**Published:** 2025-06-20

**Authors:** Farzana Rahman, Aradhana Gupta, Oluwakemi Olaoye, Nargis Uddin Amir, Pallavi Chandra

**Affiliations:** 1 Black Country Healthcare NHS Foundation Trust, West Midlands, United Kingdom; 2 Birmingham and Solihull Mental Health Foundation Trust, Birmingham, United Kingdom

## Abstract

**Aims:** Effective handovers are essential for patient safety and continuity of care. Poor communication during shift transitions is a major contributor to medical errors and adverse events. Guidelines from the Royal College of Psychiatrists (RCPsych), British Medical Association (BMA), and National Institute for Health and Care Excellence (NICE) emphasise the need for structured, distraction-free handovers with clear documentation of key clinical information.

A review of handover practices at Hallam Street Hospital, Sandwell revealed reliance on informal unregulated communication channels, primarily WhatsApp, raising concerns about confidentiality, documentation consistency, and patient safety.

This Quality Improvement Project (QIP) aimed to evaluate existing handover practices to implement a more secure and structured system.

**Methods:** A baseline survey was completed by 21 out of 35 Resident doctors (Core Trainee Year 3 and below) participating in on-call and daily handover processes. The survey assessed satisfaction, confidentiality concerns, and patient safety risks associated with the existing WhatsApp-based handover system. Findings concluded:

62% were dissatisfied with the current WhatsApp-based handover process.

66.67% felt patient safety was compromised.

61.91% lacked confidence in receiving and reading handovers by the intended recipient.

Using the Plan-Do-Study-Act (PDSA) model, the intervention involved transitioning to a structured Microsoft Teams (MS Teams) handover platform, which was already successfully implemented at Bushey Fields Hospital, Dudley.

A standardised template was produced, including key information such as patient demographics, clinical status, outstanding tasks, and risk factors. Training sessions, user guides, and drop-in support were provided to facilitate the transition.

**Results:** Post-intervention data was collected via a follow-up survey after the implementation of MS Teams Handover channel. The results demonstrated a significant improvement in handover quality:

100% of respondents were either satisfied or very satisfied with the new system.

Confidence in patient confidentiality increased, with 100% of respondents being either very or extremely confident.

Concerns regarding patient safety decreased from 66.67% to 20%.

Confidence in handovers being received and read improved significantly.

100% of doctors felt the new process met medico-legal requirements.

**Conclusion:** Transitioning from an informal Handover system to a structured MS Teams platform led to substantial improvements in documentation quality, patient confidentiality, and Resident Doctor satisfaction. The standardised approach reduced the risk of errors, improved information transfer, and aligned with national best practice guidelines. Further refinements, including optimising accessibility and ensuring sustained engagement, will be explored in future cycles of this project.

